# Genetic correlations between migraine and carpal tunnel syndrome

**DOI:** 10.1097/PRS.0000000000010976

**Published:** 2023-08-18

**Authors:** Akira Wiberg, Maria A Lucey, Sam Kleeman, Youngjoo Kang, Mike Ng, Dominic Furniss

**Affiliations:** 1 Nuffield Department of Orthopaedics, Rheumatology, and Musculoskeletal Science, University of Oxford, Oxford, OX3 7LD, UK; 2 Cold Spring Harbor Laboratory, New York, USA; 3 Department of Plastic and Reconstructive Surgery, Oxford University Hospitals NHS Foundation Trust, John Radcliffe Hospital, Oxford, OX3 9DU, UK

## Abstract

**Background:**

Surgical deactivation of extracranial nerve trigger sites is now well-established as an effective treatment for migraine headache. Parallels have been drawn to median nerve decompression for carpal tunnel syndrome (CTS), and two previous studies have demonstrated an association between migraine and CTS. We sought to: (1) substantiate these findings in a considerably larger UK cohort, and; (2) investigate potential genetic associations between the two disorders.

**Methods:**

Nested case-control studies were conducted in the UK Biobank cohort of 401,656 individuals. Odds ratios were calculated for the association between migraine and CTS in the overall cohort and sex-stratified subsets. Genetic correlation between migraine and CTS was interrogated by linkage disequilibrium score regression (LDSC), leveraging data from published genome-wide association studies. Regions of genetic overlap were identified by Multi-Trait Analysis of GWAS (MTAG) and Cross-Phenotype Association (CPASSOC).

**Results:**

Migraine and CTS show a significant epidemiological association within UK Biobank (OR=1.14, 95% CI: 1.04–1.25, p=0.0058), which is specific to females (OR=1.15; 95% CI: 1.04–1.28, p=0.0057) and not males (OR=1.07; 95% CI: 0.82–1.40, p=0.61). Genetic analysis demonstrated a significant positive genetic correlation between the two disorders (r_g_=0.13, p=0.0039), and implicated the *TRIM32* locus on chromosome 9 as a region of genetic overlap.

**Conclusions:**

This study replicates past reports of an epidemiological association between CTS and migraine, albeit in females only. This association is underpinned by a genetic correlation, with shared genetic susceptibility at the *TRIM32* locus. Our data adds credibility to the notion that an element of entrapment neuropathy underlies migraine pathophysiology.

## Introduction

Migraine headache is a disabling chronic neurologic disorder that affects 14.6% of the US population, ^1^ and is the second largest contributor to neurological disability-adjusted life years (DALYs) after stroke.^2^ It has an annual healthcare cost per patient of $11,000 USD.^3^ Pharmacotherapy for acute episodes and prophylaxis forms the mainstay of migraine treatment; however, in up to 50% of cases, medication fails to relieve symptoms or produces significant side effects.^4,5^

Surgical nerve release for migraine has emerged over the last twenty years, based on the theory that entrapment of head and neck sensory nerves trigger migraine headaches.^6^ Anatomical studies have identified four major trigger sites (frontal, temporal, occipital, and nasal), with trigger site deactivation involving one or a combination of musculofascial decompression, removal of impinging vessels, and resection of nerve segments.^6^ Histological studies support a natural history of chronic entrapment or irritation, with extracranial nerves removed from migraineurs exhibiting axonal abnormalities and dysregulated myelination.^7^ Notably, trigeminal sensory and pain fibres pass through cranial sutures between the calvarial bones.^8,9^ This provides an anatomical substrate for the intracranial transmission of peripheral triggers to meningeal nociceptors, with potential for the subsequent activation of the trigeminovascular system.

The efficacy of trigger site deactivation is appreciable: 68.3–100% of patients show at least a 50% reduction in migraine episodes, 8.3–86.5% of patients experience complete elimination of symptoms, and symptomatic improvement persists for at least 5 years post-intervention.^10–14^ Considering the anatomical, histological, and clinical evidence, migraine may be thought of as having an element of entrapment neuropathy, akin to carpal tunnel syndrome (CTS), which is treated effectively by median nerve decompression. A recent intraoperative study in migraineurs undergoing greater occipital nerve trigger site deactivation found the trapezius fascia encasing the nerve to be thickened and fibrotic in the majority of patients, similar to changes to the sub-synovial connective tissues surrounding the median nerve seen in CTS.^15^

Noting their pathophysiological similarity, Law et al. reported an epidemiological association between CTS and migraine in the US population: CTS prevalence in patients with migraine was 8% compared to 3% in those without migraine headache (adjusted odds ratio = 2.67; 95% confidence interval (CI), 2.22–3.22).^16^ Corroborating this, Gfrerer et al. found CTS prevalence to be 1.8- to 3.8-fold more common in a cohort of 137 patients who underwent surgical trigger site deactivation for migraine than in the general population.^17^ However, Law et al.’s case ascertainment question was not specific to migraine (individuals suffering “severe headaches or migraine”) and Gfrerer et al.’s study is limited by its modest cohort size and selection of surgical candidates that likely represent those migraineurs most susceptible to peripheral nerve irritability.

The present study sought to validate these findings by leveraging the power of a large UK cohort of over 400,000 individuals, and using a phenotypic definition specific for migraine. We then interrogate the genetic underpinnings of this association by using summary data from genome-wide association studies (GWAS) in migraine and CTS, and reveal a significant genetic correlation between the two disorders.

## Materials & Methods

### Ethical Approval

UK Biobank has approval from the North West Multi-Centre Research Ethics Committee (11/NW/0382). All 23andMe, Inc. research participants included in this study provided informed consent for their genotype data to be utilised for research purposes under a protocol approved by the external AAHRPP-accredited IRB, Ethical & Independent Review Services.

### Dataset

The UK Biobank prospective cohort comprises ~500,000 individuals aged 40–69 who have undergone whole-genome genotyping and linkage of these data to their medical records.^18^ We have previously conducted quality control of the raw Biobank dataset for a related study of the genetic origins of CTS, and have described this in detail elsewhere.^19^ The final cohort comprised 401,656 individuals (184,499 male, 217,157 female) of white British ancestry. Participant age was calculated on 31^st^ December 2019 based on year of birth, and participant sex was determined by genotypic rather than self-reported sex.

### Phenotyping

CTS and migraine cases were identified using diagnostic and operation codes from the UK Biobank showcase. CTS cases (n=12,312) were those with one or more of the following codes:

1. ICD-10 code for CTS (G56.0)
2. OPCS code for carpal tunnel release (A65.1) or revision of carpal tunnel release (A65.2)
3. Self-reported operation code for carpal tunnel surgery (1501)
4. Self-reported non-cancer illness code for CTS (1541)

Migraine (n=14,409) cases were those with one or more of the following codes:

1. ICD-10 code for migraine (G43)
2. Self-reported non-cancer illness code for migraine (1265)

### Epidemiological Analysis

A nested case-control study was conducted to assess epidemiological associations between CTS and migraine in UK Biobank. In this study design, cases and controls are drawn from the population of a fully enumerated cohort, and confers several advantages to a case-control study, including the ability to select cases and controls from the same underlying population, and to minimise the effect of potential confounding variables through matching.

Matching of cases to controls was performed at a 1:5 ratio using the nearest neighbour matching method in R package MatchIt,^20^ with matching variables “sex” (exact match) and “year of birth” (nearest match). In a separate set of matching, BMI (nearest match) was also included given that BMI is a risk factor for CTS and may act as a potentially significant confounder. The cobalt R package was used to assess covariate balance after matching. For the two case-control data sets (i.e. with and without BMI matching), three analyses were performed: (1) for the whole cohort; and subgroup analyses for (2) males only, and (3) females only. Odds ratios (OR) were calculated using 2×2 contingency tables with migraine as the exposure and CTS status as the outcome. Fisher’s exact tests were performed in R, and 95% CIs were calculated.

### Genetic Analysis

Summary statistics for migraine were obtained from a meta-analysis of 22 individual GWAS of around 375 000 individuals (59,674 cases, 316,078 controls)^21^ provided by the International Headache Genetics Consortium (IHGC) and 23andMe, with the latter contributing approximately half of the cases and controls to the meta-analysis (30,465 cases, 134,147 controls). Summary statistics for CTS were obtained from our previous GWAS of carpal tunnel syndrome.^19^ The genome-wide summary statistics for the two phenotypes were used to perform three independent analyses:

1. Linkage disequilibrium score correlation (LDSC) analysis: a genetic correlation (r_g_) value for CTS and migraine was calculated using LDSC v 1.0.0^22^ using the full summary association statistics. Summary statistics from CTS and migraine were standardized using the ‘munge_sumstats.py’ script in the LDSC package for python, with an INFO score threshold of 0.8 and minor allele frequency (MAF) threshold of 0.01. Genetic correlation was performed using the ‘ldsc.py’ script in the LDSC package for python, according to the authors’ tutorial^23^ using a UKB-specific LD score regression from the Pan-UK Biobank project.^24^ A p-value <0.05 indicated a significant association.
2. MTAG (multi-trait analysis of GWAS)^25^ enables joint analysis of GWAS summary statistics by combining several genetically correlated traits in order to augment statistical power and identify genomic regions of overlap. We employed this analysis with default parameters to identify genetic variants, i.e. single nucleotide polymorphisms (SNPs) that are strongly associated with both CTS and migraine. Summary statistics from CTS and migraine were standardized using the ‘MungeSumstats’ package for R. MTAG was run according to the authors’ tutorial with default parameters.^26^
3. Cross-phenotype association (CPASSOC)^27^ is a complementary analytic approach to MTAG, which also uses summary-level data from GWAS to detect variants associated with at least one trait; its statistical power is improved by analysing multiple phenotypes.^28^ Individual SNP overlaps between migraine and CTS were assessed using the beta-coefficients and standard error values for the SNPs in the two GWAS summary statistics, using the S_Het_ function in CPASSOC. We employed this analysis to identify genetic variants that were suggestive of association but not genome-wide significant in the individual CTS/migraine GWAS, but which reached statistical significance only on CPASSOC meta-analysis. We therefore conservatively prioritised SNPs with association p-values between 1×10^-5^ and 5×10^-8^ in both the migraine and CTS GWAS to avoid discovering genome-wide significant variants for the joint migraine-CTS phenotype that are driven solely by one of the phenotypes. A p-value <5×10^-8^ on CPASSOC meta-analysis indicated a significant association.

## Results

### Epidemiological Analysis

Case ascertainment within the post-quality control cohort of 401,656 individuals identified 12,312 CTS cases and 14,409 migraine cases. The matching algorithm produced two well-matched case-control datasets (Table 1).

Table 2 reports the odds ratios for the association of migraine with CTS for the whole cohort (both sexes) with and without BMI-matching, as well as for sex-stratified subgroups. Within UK Biobank, the overall odds ratio for association with CTS in migraine patients was 1.14 (95% CI: 1.04–1.25, p=0.0058). Sex-stratified analysis revealed a significant association in females (OR=1.15; 95% CI: 1.04–1.28, p=0.0057) but not in males (OR=1.07; 95% CI: 0.82–1.40, p=0.61). These data suggest that there is a significant epidemiological association between migraine and CTS in females only. Matching on BMI in addition to age and sex had the effect of marginally increasing the odds ratio in the female-specific (OR=1.17; 95% CI: 1.06–1.29, p=0.0023) and overall cohort (OR=1.15; 95% CI: 1.04–1.26, p=0.0044). Thus, despite reports in the literature of migraine and CTS both being associated with increased BMI, this was not the mediating factor.

### Genetic Analysis

Linkage disequilibrium score regression (LDSC) estimates the genetic correlation between two traits, with a correlation coefficient ranging from –1 to +1. The LDSC analysis between the CTS and migraine GWAS summary statistics yielded a statistically significant positive genetic correlation coefficient of 0.13, Z-score=2.89, p=0.0039, providing strong evidence for a shared genetic architecture between CTS and migraine underlying the epidemiological association.

**Figure 1:**
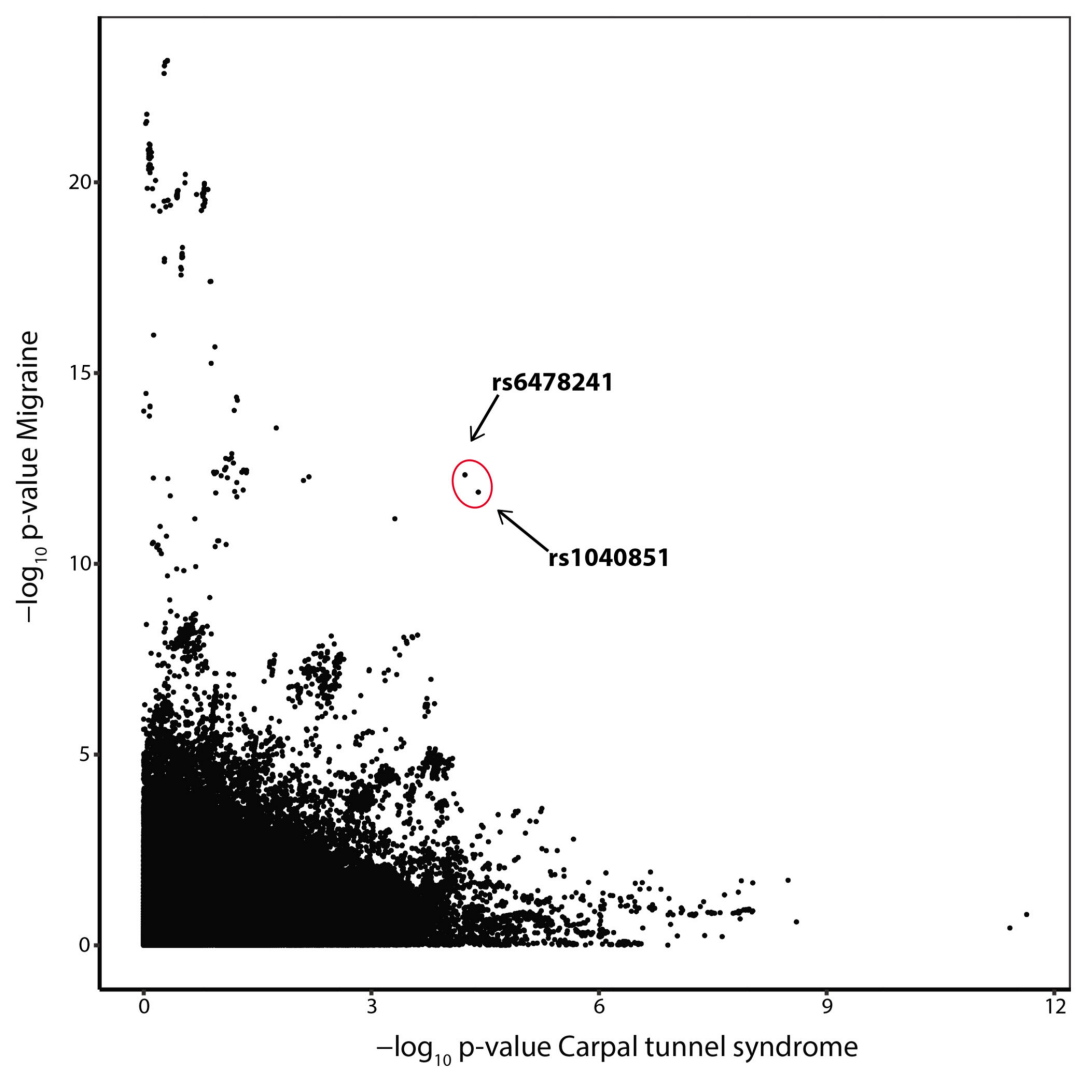
Multi-trait analysis of GWAS (MTAG) analysis. Output of MTAG meta-analysis, demonstrating two single nucleotide polymorphisms (SNPs), rs1040851 and rs6487241 on chromosome 9, that are clear visual outliers when the strength of association with migraine (y-axis) is plotted against CTS (x-axis).

MTAG analysis, which jointly analyses genetically correlated traits, demonstrated two single nucleotide polymorphisms (SNPs) on chromosome 9 – rs1040851 and rs6487241 – that were clear visual outliers when their strength of association with both migraine and CTS were plotted following meta-analysis (Fig. 1). For both SNPs, the strength of the statistical association with both phenotypes increased on MTAG meta-analysis (Table 3). rs1040851 is strongly correlated with rs6478241 (r^2^=0.87 in British ancestry populations, p<0.0001), and both lie approximately 200kb downstream of, and are expression quantitative trait loci (eQTL) of the *TRIM32* gene,^29^ meaning that these variants alter the expression levels of this gene.

**Figure 2:**
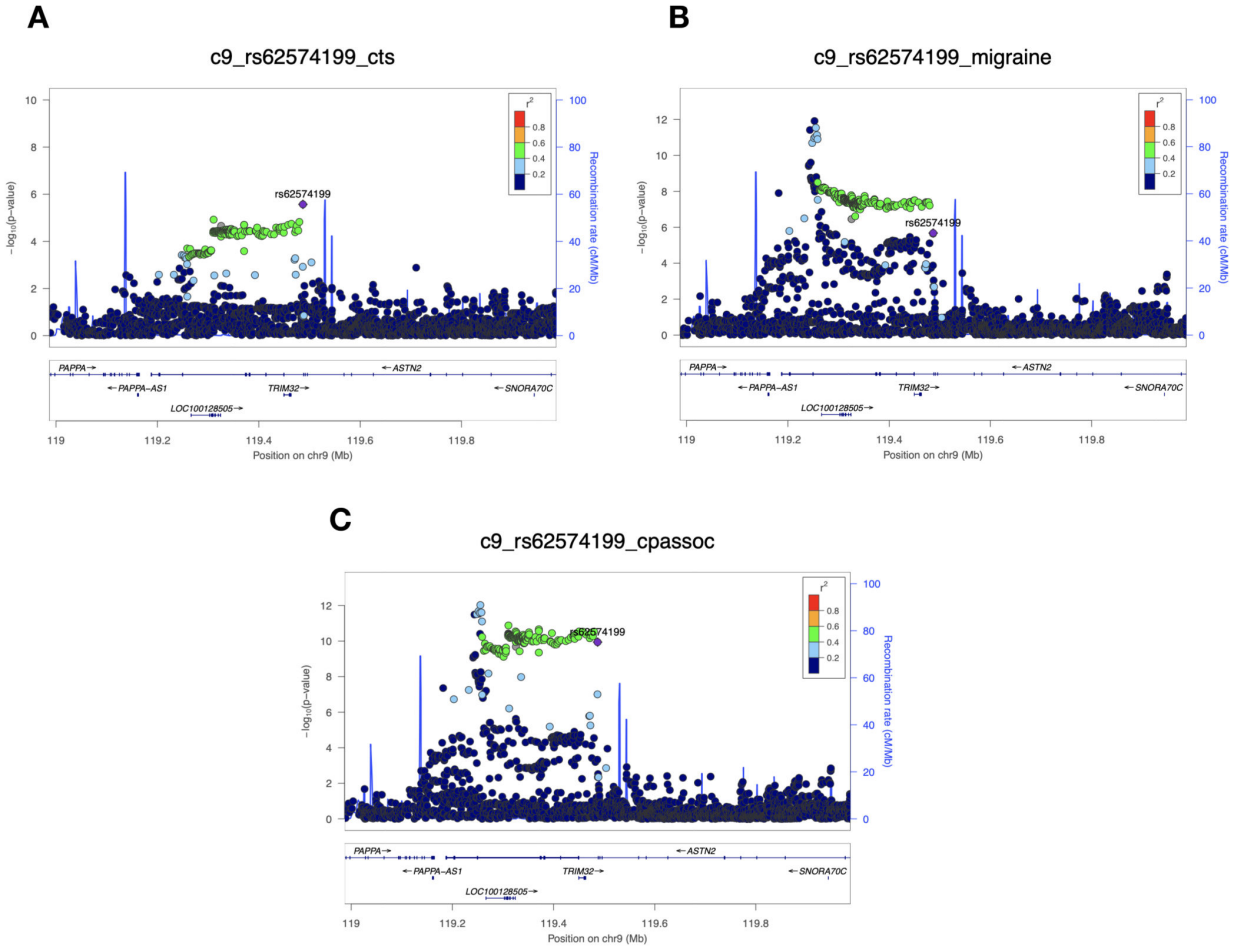
Cross-phenotype association (CPASSOC) analysis. LocusZoom plots of the variant rs62574199, showing its association with carpal tunnel syndrome (panel A) and migraine (panel B) in their respective GWAS, and with the joint CTS-migraine phenotype on CPASSOC meta-analysis (panel C). rs62574199 is shown in purple, and each of the circles represents a single nucleotide polymorphism (SNP). The degree of correlation (linkage disequilibrium) between the SNPs and rs62574199 is demonstrated by colour-coding (inset, top right). The x-axis indicates the genomic position on chromosome 9, with genes in the region shown, including *TRIM32*.

Our complementary analytic approach, CPASSOC, corroborated the importance of this genomic region in terms of CTS-migraine overlap; there was just a single SNP that satisfied the pre-determined p-value criteria of having a suggestive association in the individual GWAS of CTS and migraine, and reaching genome-wide significance only on meta-analysis: rs62574199. This SNP is an intronic (i.e. non-coding) variant in the gene *ASTN2* (Fig. 2), and although it is not strongly correlated with rs1040851 (r^2^=0.09, p<0.0001), it is also an eQTL of *TRIM32*, and therefore also affects the expression of this gene. The two results, taken together, suggest the importance of *TRIM32* in mediating the shared genetics between CTS and migraine.

## Discussion

Anatomical, histological, and clinical evidence accumulated over the past two decades strongly suggests that migraine exhibits features of a nerve entrapment disorder that may be successfully treated by surgical trigger site deactivation. Two previous studies have reported an epidemiological association between migraine and CTS, the quintessential entrapment neuropathy. The present study sought to test the veracity of this association in a substantially larger cohort of 401,656 individuals, as well as to leverage GWAS data to investigate the genetic foundations of this association.

A clear epidemiological association between migraine and CTS was found in the UK Biobank cohort in this study (OR 1.14, 95% CI: 1.04–1.25, p=0.0058). Sex-stratification revealed that this association was specific to females (OR 1.15, 95% CI: 1.04–1.28, p=0.0057), being non-significant in males (OR 1.07; 95% CI: 0.82–1.40, p=0.61). Whilst both migraine and CTS are associated with raised BMI,^30,31^ this was not a mediating factor, as matching cases and controls on BMI had little material effect on the strength of association in both the overall and female-specific cohorts.

Comparing the present findings with previous studies, both Law et al. and Gfrerer et al. report a notably stronger association between migraine and CTS, between 1.8 and 3.8-fold. Differences in inclusion criteria likely explain this discrepancy. Law et al.’s use of a broad case definition of migraine (“migraine or severe headache”) led to a self-reported diagnosis of migraine in 16.3% of respondents, whereas only 3.6% (14,409) of individuals had a migraine diagnosis in our UK Biobank cohort. The proportion of CTS cases was similar between the studies (3.7% and 3.1%, respectively), and thus the large difference in odds ratios between the studies is likely attributable to differences in the ascertainment of migraine cases. In contrast to Law et al.’s broad inclusion criteria, Gfrerer et al. only included patients who had undergone trigger site deactivation surgery for migraine, which may have enriched for migraineurs who are susceptible to entrapment neuropathy.

The case ascertainment for migraine and CTS in our study also had its limitations. Firstly, our case/control classification was based on hospital diagnostic codes, self-report, or both. Individuals who we designated as cases based on self-report alone are more likely to have been misclassified than individuals with a hospital diagnostic code. Even the latter group may have been misclassified – for instance, not all individuals with a CTS hospital diagnostic code will have had confirmatory electrodiagnostic studies. Secondly, given that our dataset did not include diagnostic codes from primary care (family physicians) – where the majority of healthcare delivery in the UK occurs^32^ – a proportion of true cases will have been misclassified as controls, thereby underestimating the true population prevalence of both diseases. Thirdly, our study was limited to individuals of white British ancestry to avoid confounding through the variable prevalence of migraine/CTS in different ancestry groups; trans-ancestral analysis of the migraine-CTS association will be a focus of future studies.

Despite the discrepancies in the reported odds ratios of migraine-CTS co-occurrence across the three studies, what is consistent is the existence of a statistically significant epidemiological association between migraine and CTS.

Analysis of the genetic overlap between migraine and CTS by linkage disequilibrium score regression indicated a strong genetic correlation, with a positive genetic correlation coefficient (r_g_) of 0.13. By way of comparison, a recently published study substantiating a genetic correlation between migraine and blood pressure (for which an epidemiological relationship has been reported^33^) found lower r_g_ values than that reported here.^34^ To our knowledge, we report the first demonstration of a genetic association between migraine and CTS, and this bolsters the epidemiological association reported in our study and others.

Scrutinising the loci of genetic overlap from our two complementary genetic analyses, a region on chromosome 9 was found, implicating the gene *TRIM32*. We identified at least three SNPs in the vicinity of this gene that show the strongest association with both migraine and CTS, all of which alter the expression levels of *TRIM32*. *TRIM32* encodes human tripartite motif family of proteins 32 (TRIM32), a ubiquitous multifunctional protein that has roles in muscle homeostasis, glucose metabolism, and both tumour suppression and tumourigenesis.^35^ Neurons from the brains of *TRIM32* knockout mice have reduced neurofilament protein expression, suggesting a role for TRIM32 in neuronal maintenance.^36^ As extracranial nerves isolated from migraineurs exhibit neurofilament disruption and dysregulated myelination^7^, TRIM32 may be tentatively implicated in entrapment neuropathies via a role in neuronal maintenance. Of note, a recent study reporting a highly significant genetic correlation between endometriosis and migraine (r_g_=0.38, p=2.30×10^-25^) also implicated *TRIM32* as an overlapping gene between the two disorders.^37^ The role of *TRIM32* as a potential risk variant in migraine, CTS, and endometriosis is yet unclear and requires further study, though it is intriguing to note its association with three disorders that predominantly affect females.^38–40^

The notion of migraine as a peripheral nerve disorder remains debatable, as it conflicts with longstanding theories of central generation of migraine. However, and consistent with a polygenic model of migraine susceptibility^21^, it may be that only a subset of migraineurs have peripheral aetiology, and this may be the subgroup who benefit from trigger site deactivation surgery.^41^ Accepting this does not devalue the contribution of central mechanisms (trigeminovascular system activation and sensitisation, cortical spreading depression, parasympathetic input) to migraine generation, and medications targeting these events will continue to be critical to treatment, alongside surgical trigger site deactivation in selected patients. Ideally, our study would have been able to stratify patients with peripherally-generated migraines to examine the strength of association of CTS with this subset of migraine alone; however, surgery for migraine is not yet routinely undertaken in the UK, so we were unable to make the distinction between surgical and non-surgical migraine patients based on the dataset available to us.

In summary, we provide the first ever demonstration of a significant genetic association between migraine and CTS, suggesting shared susceptibility or pathophysiology. We also validate the previously related epidemiological association between the two disorders in a substantially larger cohort. CTS is the archetypal entrapment neuropathy that is successfully treated by surgical decompression. By demonstrating the genetic underpinnings to the epidemiological association between migraine and CTS, our findings add further credibility to the idea that migraine pathophysiology may be in part mediated by peripheral nerve entrapment or a triggering mechanism, thus providing a new lens for considering the value of migraine surgery.

## Figures and Tables

**Table 1:** Characteristics of the case-control cohorts. This table shows the total number of individuals, mean BMI, mean age, sex distribution, and numbers of individuals with CTS for (1) the migraine cases, (2) the non-migraine controls (pre-matching), and (3) the non-migraine controls (post-matching). Matching of migraine cases to controls was performed at a 1:5 ratio, using the R package MatchIt.

	N	Mean BMI (SD)	Mean Age (SD)	males (%)	females (%)	CTS (%)
**Migraine Cases**	14,409	27.1 (5.0)	65.6 (7.8)	3,368 (33.4)	11,041 (76.6)	551 (3.82)
**Controls (pre-matching)**	387,247	27.3 (5.0)	66.9 (8.0)	181,142 (46.8)	206,105 (53.2)	11,761 (3.04)
**Controls (post-matching)**	72,045	27.2 (5.0)	65.6 (7.8)	16,840 (33.4)	55,205 (76.6)	2,424 (3.36)

**Table 2:** Odds ratios for having a CTS diagnosis in the two nested case-control cohorts. The “BMI” suffix refers to matching on body mass index as an additional matching variable in addition to year of birth and sex.

DISEASE		Odds ratio of CTS (95% CI)	Z-statistic	P-value
**Migraine**	Whole cohort	1.14 (1.04–1.25)	2.76	0.0058
	Males	1.07 (0.82–1.40)	0.51	0.61
	Females	1.15 (1.04–1.28)	2.77	0.0057
**Migraine_BMI**	Whole cohort	1.15 (1.04–1.26)	2.85	0.0044
	Males	1.01 (0.77–1.31)	0.045	0.96
	Females	1.17 (1.06–1.29)	3.05	0.0023

**Table 3:** Association p-values for the single nucleotide variants rs1040851 and rs6478241 with migraine and CTS, before and after meta-analysis using MTAG.

	rs1040851		rs6478241	
	Pre-MTAG	Post-MTAG	Pre-MTAG	Post-MTAG
**P-value (CTS)**	1.40×10^-3^	3.87×10^-5^	0.0063	5.80×10^-5^
**P-value (Migraine)**	3.86×10^-12^	1.32×10^-12^	1.22×10^-12^	4.67×10^-13^
